# Institutional Housekeeping

**Published:** 1912-03-23

**Authors:** 


### INSTITUTIONAL HOUSEKEEPING.

(Workers' Questions and Criticism of every kind will be welcomed.)

### The Wastefulness of Boiling.

It is heartrending for the economist to consider how frequently his best efforts are neutralised in the kitchen. Among all the sources of loss which the hospital sustains perhaps none is more glaring than what goes on during the common process of boiling. To the uninstructed woman the process of boiling means putting food into water and setting it over the fire for a given length of time, according to its bulk. The result is sent to table and consumed with more or less dissatisfaction, none of the parties to the transaction being aware that the properties which ought to be nourishing their frames are peacefully reposing in the sink until someone has time to throw them away, and that the meat which they consider the foundation of the meal has been drained of its value by the process which prepared it for consumption.

Few untrained cooks realise that boiling has two distinct and widely different objects, the one to retain nutritive juices and flavour, and the other to extract them. They go astray in both processes, but more disastrously in the former than in the latter.

In the majority of boiling operations, the term used is really an error of language. It is confusing to those who use it, because taken literally it would mean absolute failure. For instance, to boil custard would result in the coagulation and separation of the egg albumen, and the result would be a rough curdled mixture. Again, to boil meat by allowing it to remain in water at the boiling point of 212° results in the coagulation and hardening of the albumen throughout the joint, and makes it uneatable.

In order to retain the nutritive qualities of meat and poultry and render it well-flavoured and juicy under the boiling process, the joint must be plunged at once into boiling water, 212° Fahr., so as firmly to coagulate the albumen on the outside. This forms as it were an envelope which, partially, at any rate, protects the inside of the meat from the solvent action of the water. The temperature must be kept at boiling-point for five or ten minutes, after which time the water should be cooled down to about 180° Fahr., to prevent the albumen throughout the joint coagulating also, which would make it tough and hard. In carrying out these processes, the use of a kitchen thermometer is indispensable. The neglect of this precaution, as we have already pointed out, is responsible for much discreditable cooking and much unfair blame of the butcher.

But, however much care is applied to the process of cooking meat in water, a large amount of waste is inevitable. True, by plunging the meat in boiling water to start with, its juices are to a great extent retained, but some will undoubtedly be lost. Thus, one authority states that a loss of one pound in every four may be expected in the boiling process. Various kinds of apparatus have been constructed for the purpose of economising fuel, while rendering possible the long, slow cooking of foods in moderate heat which most conduces to their digestibility. The simplest of all these is constructed on the double pan system, the food being placed in the inner cooker, the water in the outer vessel being kept at or near boiling-point. In order to reduce the waste of extractives and salts as much as possible the pan should be only just sufficiently large to hold the food, and no more water used than is necessary nearly to cover the joint or other article of food. The larger the proportion of water to the meat, the greater naturally will be the loss of soluble substances.

In boiling fish the same process is to be followed as in boiling meat or poultry—namely, to plunge in boiling water to coagulate the albumen and so retain the juices, and then to subject to slow heat. But as the flavouring substances of fish are even more easily dissolved and lost than those of meat, and the actual flavour is far more delicate to start with, greater care is needed. In preparing fish for invalids the double pan process is greatly to be preferred to ordinary boiling, as it is essential their food should not be deprived of any of its nourishing qualities. The loss which fish undergoes in the boiling process may be estimated by a study of the following table, given by Miss K. Williams in the Journal of the Chemical Society:—

Boiled Fish as Served at Table.

| Variety | Part Analysed | Waste: Bones, etc. | Gelatin | Water | Nutrients |
|---|---|---|---|---|---|
| Herrings ... | Whole | 11·74 | 0·63 | 52·99 | 34·54 |
| Whiting ... | Whole | 21·50 | 0·86 | 61·29 | 16·35 |
| Plaice ... ... | Flesh only | — | — | 79·86 | 20·14 |
| Sole ... ... | Whole | 22·02 | 0·74 | 61·18 | 16·06 |

It will be seen from what has been said that for institution use, under hurried or imperfectly trained cooks, boiling is very risky. At worst, the boiling process may render the meat almost uneatable by hardening fibres, or it may wash out the principal part of the soluble substances into an excess of water subsequently thrown away. It must at best reduce the nutrient properties by about one-fourth, although by softening the indigestible fibrous framework of the tissues it renders the access of gastric juice easier to the remainder. And unless skilful flavouring be added the result will be flat and unappetising. In many large institutions meat and fish are cooked in steamers, that is, in the vapour from boiling water, not in water itself. If the pressure is correctly maintained condensation is extremely slight, and the waste is reduced to a minimum. On this account this method is greatly to be preferred to the old-fashioned one of cooking in water.

